# Establishing a governance threshold in small-scale fisheries to achieve sustainability

**DOI:** 10.1007/s13280-021-01606-x

**Published:** 2021-08-17

**Authors:** Alba Aguión, Elena Ojea, Lucía García-Flórez, Teresa Cruz, Joxe Mikel Garmendia, Dominique Davoult, Henrique Queiroga, Antonella Rivera, José Luis Acuña-Fernández, Gonzalo Macho

**Affiliations:** 1 Future Oceans Lab, CIM-Universidade de Vigo, Torre CACTI, Campus Lagoas Marcosende, 36310 Vigo, Spain; 2 Centro de Experimentación Pesquera, Dirección General de Pesca Marítima del Principado de Asturias, 33212 Gijón, Spain; 3 MARE - Marine and Environmental Sciences Centre, Laboratório de Ciências Do Mar, Universidade de Évora, Apartado 190, 7521-903 Sines, Portugal; 4 Departamento de Biologia, Escola de Ciências E Tecnologia, Universidade de Évora, 7002-554 Évora, Portugal; 5 AZTI - Unidad de Investigación Marina, Herrera Kaia, Portualdea, 20110 Pasaia, Spain; 6 Sorbonne Université, CNRS, UMR 7144 AD2M, Station Biologique de Roscoff, 29680 Roscoff, France; 7 Departamento de Biologia and CESAM - Centro de Estudos Do Ambiente E Do Mar, Universidade de Aveiro, 3810-193 Aveiro, Portugal; 8 The Coral Reef Alliance, Mesoamerican Region, 1330 Broadway, Suite 600, Oakland, CA 94612 USA; 9 Observatorio Marino de Asturias, Departamento de Biología de Organismos Y Sistema, Universidad de Oviedo, Calle Valentín Andrés Alvarez, 33006 Oviedo, Spain; 10 Estación de Ciencias Mariñas Illa de Toralla (ECIMAT), Universidade de Vigo, 36331 Vigo, Spain; 11 Independent Fisheries Consultant, Fisherman’S Cove, Seychelles

**Keywords:** Co-management, Governance, Small-scale fisheries, Sustainability

## Abstract

The lack of effective governance is a major concern in small-scale fisheries. The implementation of governance that encompasses the three pillars of sustainability (social, economic, and ecological) is still a worldwide challenge. We examined nine stalked barnacle fisheries (*Pollicipes pollicipes*) across Southwest Europe to better understand the relationship between governance elements and sustainability. Our results show that nested spatial scales of management, the access structure, co-management, and fisher’s participation in monitoring and surveillance promote sustainability. However, it is not the mere presence of these elements but their level of implementation that drives sustainability. Efforts should be placed in the accomplishment of a minimum combination of local scales of management, access rights through individual quotas, instructive-consultative co-management and functional participation. Surpassing this threshold in future governance structures will start to adequately promote social, economic and ecologically sustainability in small-scale fisheries.

**Supplementary Information:**

The online version of this article at 10.1007/s13280-021-01606-x.

## Introduction

One of the major goals of sustainability science is the implementation of governance structures that enhance human well-being through sustainable ecosystem use. For effective governance, a solid understanding of the interactions between humans and the ecosystem on which they rely on is needed (Carpenter et al. 2009). However, fisheries worldwide are often characterized as unsustainable and are typically governed under structures that have failed to reverse negative trajectories (Pauly et al. 2002; Worm et al. 2009). Shifts towards more appropriate governance settings might dramatically improve the situation of global fisheries (Costello et al. 2016), offsetting productivity changes of future threats like climate change (Gaines et al. 2018). However, there is little global evidence on how governance can embrace the ecological and social constituents of ecosystems and their interactions to promote sustainability (Carpenter et al. 2009), denoting that fisheries are not alone in the struggle.

Traditionally, fisheries management has focused on one or few species, ignoring habitat, governance and other ecosystem components and their interactions. Governance has been historically based on the establishment of rules by central governments, who depend on enforcement options to achieve compliance. Although this governance might solve certain problems (i.e., overfishing of a stock), it is unable to deal with cumulative stressors or adequately link social and ecological processes (Crowder et al. 2006), failing to provide incentives to users (Beddington et al. 2007). The continuous challenges faced by fisheries have prompted the arise of new governance arrangements focused on the allocation of incentives and the creation of shared knowledge, fostering the establishment of partnerships between government and users (Basurto et al. 2017). In this context, governance has evolved to recognize the importance of the human dimension in the management of natural resources (Ostrom 2009; Cohen et al. 2019). The links established among stakeholders, the involvement of fishing communities in decision-making or the way fishers are granted access to the resource are considered in novel governance settings besides the traditional top-down rules (e.g., quotas, closures or catch and effort controls) (Symes 2006). In this paper, the definition of governance extends to all principles that determine the behaviour of users in the harvesting activity, capturing the importance of the social aspect in fisheries management.

The lack of effective governance in small-scale fisheries is a major concern of the Food and Agriculture Organisation (FAO) of the United Nations (FAO 2015). Small-scale fisheries account for about half of the world fish catch (two-thirds when only considering catches destined for direct human consumption) and employ over 90% of fishers involved in capture fisheries (World Bank 2012). However, small-scale fisheries have historically been unaccounted for, underestimated and hidden within national statistics (Pauly and Charles 2015; Smith and Basurto 2019). This undervaluation hinders the recognition of dysfunctional status within the sector which, is key in transformational changes towards effective governance (Gelcich et al. 2010). The problem is also exacerbated by the intrinsic heterogeneous set of social-ecological interactions in small-scale systems, that calls for tailored interventions at detailed geographic scales (Leslie et al. 2015).

In this context, small-scale fisheries targeting sedentary and low mobility resources (also known as *S-fisheries*) have played a key role in the development of novel governance arrangements (Orensanz et al., 2005; Orensanz and Seijo 2013; Defeo et al. 2016). This capacity to evolve novel solutions is mainly related to their heterogeneous spatial structure and the spatially restricted impacts of fishing effort in their populations. This spatial complexity requires a high level of spatial detail in monitoring (e.g., resource assessment) and surveillance (e.g., checks for enforcement) (Fernandez-Boan et al. 2013), often too costly or technically unachievable. As an alternative, some of the fisheries targeting spatially structured stocks have focused on governance settings that provide fishers’ incentives to achieve compliance (Orensanz et al., 2005). Several of these fisheries have redesigned the top-down governance model to successfully incentivize fishers in Latin America (Orensanz and Seijo 2013; Defeo et al. 2016), Australia (Prince 2010; Gilmour et al. 2013), Japan (Yamamoto 1995; Uchida and Machino 2008) and Europe (Symes et al. 2003; Gutiérrez 2015).

However, the small-scale fisheries that have successfully shifted towards novel bottom-up arrangements differ in the combination and extent of their governance elements. Co-management is a governance element suggested to enable the collaboration across diverse stakeholders, develop new knowledge and increase the capacity of the system to deal with new drivers (Defeo et al. 2016; d´Armengol et al. 2018). Although usually treated under a presence or absence approach, fisheries co-management covers a spectrum of levels that range from minor signs of decentralization to the delegation of authority to users (Table S4) (Sen and Nielsen 1996). The access structure of fisheries is another governance element eligible to promote sustainability (Orensanz and Seijo 2013; Costello et al. 2016), through a sense of ownership that benefits conservation and tackles the tragedy of the commons (Hardin 1968). But similarly, the way individuals are allowed access to the resource covers a variety of levels that range from open access to exclusive property rights like Territorial User Rights for Fishing (TURF), with different limited entry categories between them (Table S6) (Hilborn et al. 2005). Different combinations and implementation levels of governance elements like co-management and the access structure coexist in benthic small-scale fisheries worldwide, triggering their particularly heterogeneous set of governance and rules-in-use (Basurto et al. 2013). Given their multiple social-ecological interactions, it is difficult to independently associate governance elements to a particular sustainability level. Hence, no study has so far compared the performance along the governance spectrum of small-scale fisheries from a broad social-ecological perspective.

In this work, through the study of the heterogeneous governance of a sedentary resource (stalked barnacles) across Southwest Europe, we assess the implications of governance elements implemented at different extents in the social-ecological sustainability of fisheries. As far as we know, our study represents the first comparison of governance approaches and sustainability levels across the commercial distribution area of a coastal resource. We study the four main governance elements when managing complex small-scale fisheries: the spatial scale at which regulations are set and data collected, the responsibilities of fishers and authorities in decision-making (co-management), the level of fishers participation in control, monitoring and surveillance and the way fishers are allowed access to the resource (access structure) (see description and sources in Methods). Fisheries sustainability is assessed through the presence of a set of 19 attributes previously recognized to promote long-term sustainability from a socioeconomic and ecological perspective (Gutiérrez et al. 2011, see Table 2). A better understanding of the interrelations among governance elements and their outcomes enables the development of a general model for governing small-scale resources.

## Materials and methods

### The stalked barnacle fisheries

The European stalked barnacle fisheries have an annual economic value of EUR 10 million, with around 500 t of landings and 2100 professional fishers involved. The main fishery is in Galicia, with over half of the landings and value (Table 1). Fishers usually combine their extraction with other small-scale resources (such as octopus, crabs and coastal fish) or another economic activity, although a significant number of fishers that live exclusively on the resource has been reported in Galicia (own expertise) and in a natural park in Alentejo-Algarve (PNSACV) (Carvalho et al. 2017).

**Table 1:**
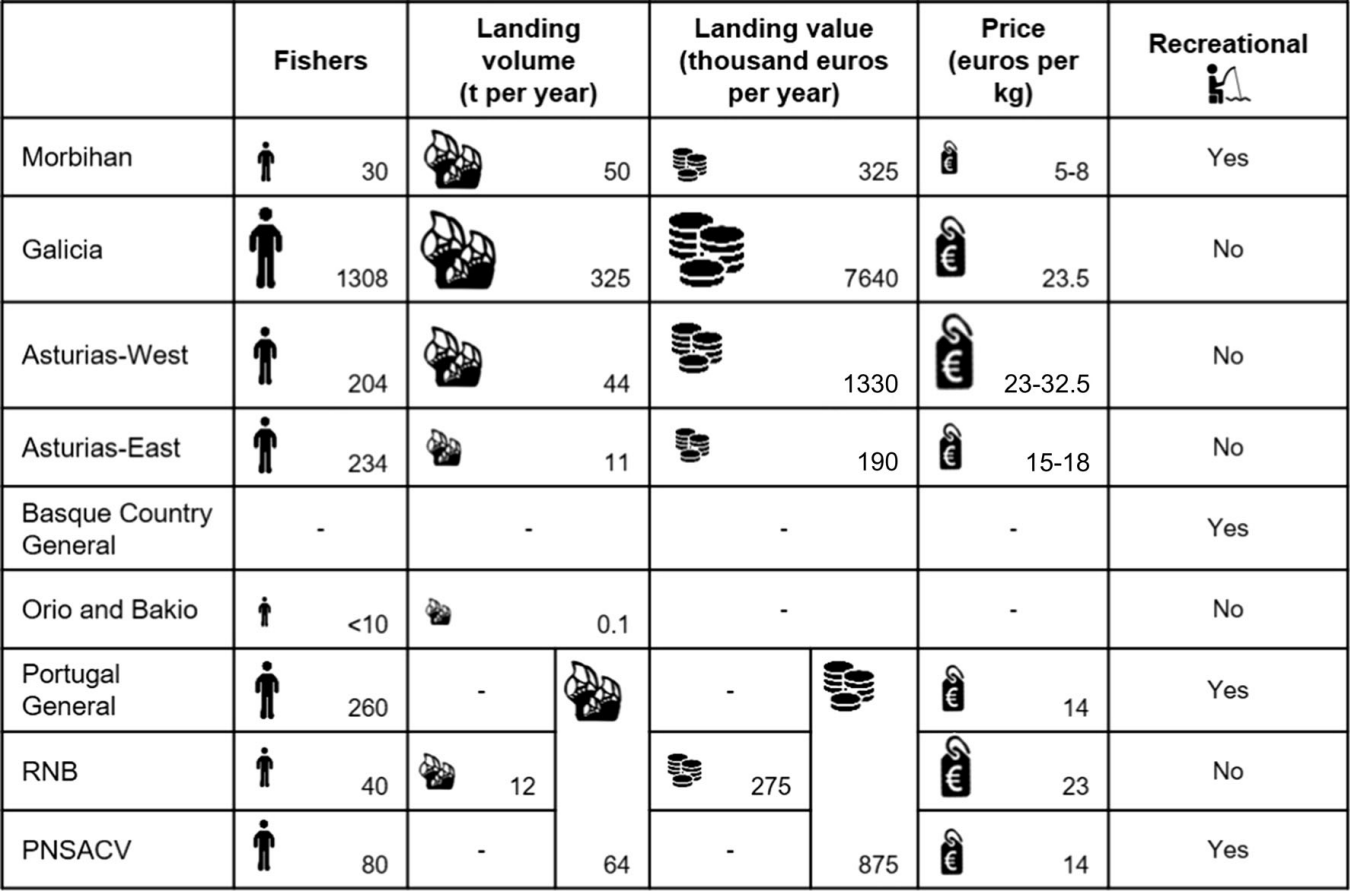
Socioeconomic characterization of the stalked barnacle fisheries in SW Europe. Number of fishers, landings (volume and value), ex-vessel price (average values for the period 2013–16^1^ are given) and the presence of recreational fisheries in each area. The “- “ symbol indicates that the data was unavailable for the fishery. The icons reflect the qualitative level of each variable, as they are not exactly proportional to the numbers, and are meant for visualization purposes

^1^Data belongs to 2013–2016, except RNB, PNSACV and data for the combination of the different Portuguese fisheries. See complete description and data sources per fishery in Table S2 of the SSMM

**Fig. 1:**
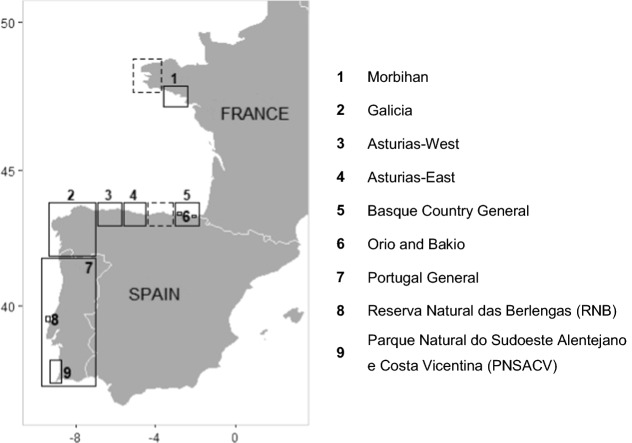
The nine European stalked barnacle fisheries included in this study. Dotted squares represent the location of the Finistère (France) and Cantabria (Spain) fisheries, not included in this work due to the lack of data. No fishery occurs from Morbihan until the north of Spain as stalked barnacles are practically absent due to the dominance of sandy shores between these regions. Map coordinates are in decimal degrees

We define a stalked barnacle fishery as a specific area with a common set of policies implemented to regulate the commercial and, if present, the recreational harvesting of the resource. Following this definition, 11 stalked barnacle fisheries were identified and nine of them are subjects of this study (Fig. 1). While in France and Portugal national administration bodies are in charge of the management, the Spanish Central Administration has delegated this responsibility to the Autonomous Communities (Galicia, Asturias and the Basque Country in our study) (Table S1). In Galicia and Asturias-West, the fisheries are based on management plans spatially allocated to fishers’ associations (locally known as *cofradías*). In the Basque Country, two small management plans (Orio and Bakio) have recently been established. In the rest of the Basque Country, Morbihan, Asturias-East and Portugal, barnacles are managed through general regulations without management plans. However, in Portugal there are specific regulations for the fishery in two areas: the natural reserve of Berlengas (RNB) and a natural park in Alentejo-Algarve (PNSACV).

### Governance elements

**Fig. 2:**
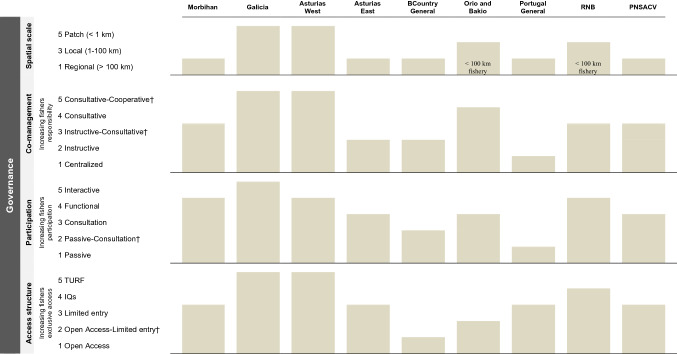
Levels of the governance elements per stalked barnacle fishery in SW Europe. Includes spatial scale of management, co-management, fisher’s participation and access structure. New intermediate levels were created to original levels defined in S4, S5 and S6^†^

Four elements of governance were analyzed in each fishery: the spatial scale of management, co-management, the access structure and the participation of fishers. The first three are the primary elements of governance identified by Hilborn et al. (2005) after the study of a range of institutional structures across fisheries. We have also included fisher’s participation in control, monitoring and surveillance due to the importance many authors have given to this element in spatially structured fisheries like barnacles (Parma et al. 2003; Orensanz et al. 2005; Prince 2010; Dias et al. 2020). Due to the scarcity of data in these fisheries, the participation of fishers in data collection and assessment is suggested to be the only way to obtain information at the appropriate scale for meaningful management (Parma et al. 2003). Although co-management and fisher’s participation are governance elements particularly interrelated, enough differences between the involvement of fishers in decision-making (co-management) and their participation in the monitoring, control and surveillance (fisher’s participation) exist to consider them separately (see Fig. 2). We acknowledge that there are other elements relevant in fisheries governance (equity and equality, human rights and dignity, etc.), but a comprehensive analysis of all possible elements was beyond the scope of this paper so we focused on the most relevant ones for our case study.

For each governance element we assigned one-unit increasing values starting from 1 to their different levels, except for the spatial scale where each level increases by two units (see description of each level in Tables S3 to S6 in SSMM and values assigned in Fig. 2). The values of the four governance elements were summed to obtain a governance score per fishery. To give the same weight to the four elements in the final score, all elements were ranked between 1 and 5. While a governance score of four is the minimum (top-down governance), 20 is the maximum. We considered fisheries that scored seven or higher to be subjected to bottom-up settings.

#### Spatial scale of management

To analyze the spatial scale at which regulations are set, three relevant scales were identified: regional, local and patch (Orensanz et al. 2016). These scales are broad as they are focused on biological processes of interest (from connectivity by larval dispersal to density dependent processes), where dimensional bounds change depending on the species (Table S3). In the larger scale (regional), disjointed populations are connected through larval dispersal (Hilborn et al. 2005). The typical larval dispersal of stalked barnacles has been estimated to be 100 km (Table S3), so regionally managed fisheries are considered those whose regulations are set above that distance. The intermediate scale (local) corresponds to the scales of fishing beds (in the scale of 10 s kilometers, but below the larval dispersal distance). The finest spatial scale (patch) corresponds to small portions of the fishing bed, the typical scale used by researchers to conduct experiments. We have considered that patch spatial scale is present when fisheries management has the capability to operate at the level of neighborhoods of individuals, at the scale of a rock/s, so around one km. We investigated if there is a regional, local or patch scale across fisheries, assigning scores of 1, 3 or 5 respectively.

#### Co-management level

To analyze the level of responsibility of the government and fishers in decision-making we used the Sen and Nielsen (1996) classification. These authors defined different co-management types that range from minimal collaboration to delegation of authority to users (Table S4). As co-management increases across the scale, rights of users in decision-making are expected to be higher. When harvesters do not have any type of responsibility in the management of the resource at any stage of the process, co-management is absent, and thus, we considered fisheries to be subjected to a centralized management. See values assigned for each co-management level in Fig. 2.

#### Fisher´s participation

To evaluate the form, extent and impact of the participation of fishers in the monitoring, control and surveillance of the fishery we used the Pretty (1995) scale, ranging from pseudo-participation to increasing levels of genuine participation (Table S5). See values assigned for each level of fisher’s participation in Fig. 2.

#### Access structure

To analyze the way fishers are allowed access to the resource across fisheries we used Hilborn et al. (2005) classification. This scale ranges from open access fisheries to the most exclusive form of access, TURF. At this scale, the upper levels include the attributes of the levels below, increasingly adding aspects that bring a higher level of exclusivity to users (Table S6). See values assigned for the different access structure levels in Fig. 2.

### Sustainability assessment

The long-term sustainability of the fisheries was analyzed from an ecological and socioeconomic perspective. To compare the fisheries’ sustainability, we used the 19 attributes identified by Gutiérrez et al. (2011) in the governance, users and resource system of fisheries (based on Ostrom 2009) (Table S7). When more than eight attributes were present the authors found a strong positive relationship, with increasing attributes leading to higher success scores. Based on this, we considered fisheries to have poor sustainability levels when less than eight attributes were present, coinciding with the two lowest success score categories defined by the authors (see Figs. 1 and 2 in Gutiérrez et al. 2011). Fisheries that ranged from eight to eleven attributes were considered to have middle sustainability levels, while higher sustainability was recorded when 12 or more attributes were present, coinciding with the two highest success scores categories (Gutiérrez et al. 2011).

### Data compilation and statistical analysis

To explore the European stalked barnacle fisheries, we searched in the literature for socioeconomic data, technical regulations and governance information. To fill the numerous gaps, additional information was collected through consultation with local experts from the administration and the scientific community. Our own personal knowledge of the fishery was also used. In the PERCEBES project (PCIN-2016–120; EU Horizon 2020 BIODIVERSA- ERA-2015) closing meeting (January 2020), a stakeholder consultation was held with fishers, managers and scientists. During the workshop, a cross check of the sustainability attributes and governance settings was done to keep relative consistency between fisheries.

A linear regression analysis between the governance score and the number of sustainability attributes was conducted to explore the strength of association between them. The principal assumptions that justify the use of linear models were met: linearity, independence. homoscedasticity and normality. The analysis was done using R statistical software (www.r-project.org).

## Results

### Governance elements

#### Spatial scale of management

We found that barnacle management ranges from fisheries that set regulations at regional, local and patch spatial scales (Galicia and Asturias-West), to fisheries that only have a common regional scale (Morbihan, Asturias-East, Basque Country, Portugal and PNSACV) (Fig. 2). In the fisheries of Galicia and Asturias-West, although there is a general regulation framing management for the whole region, smaller areas are spatially allocated to fishers’ associations (local scale) (which usually have between 10–60 km of coastline). There are 37 management plans in Galicia commonly divided in subzones (2–10 km) (Pesca de Galicia, 2019) within which some specific rocks are most of the time closed and are only harvested in particular moments. In Asturias-West the eight management plans are subdivided into 250 zones according to resource quality (zones can be as small as 3 m), and catch monitoring is done at this micro/patch scale (Rivera et al. 2014). In the other barnacle fisheries where local management was identified (Asturias-East, Orio and Bakio and RNB), the patch scale was absent, and as their coast length is below the typical larval dispersal distance, the regional scale of management does not apply.

#### Co-management level

A spectrum of responsibility levels for fishers was found, ranging from completely absent (Portugal General) to fisheries actively involved in decision-making throughout an intermediate level between consultative and cooperative co-management (Galicia and Asturias-West) (Fig. 2). In the *cofradías* of Galicia and Asturias-West, fishers usually lead daily decisions such as changing to another patch for harvest, reducing the daily quota or stopping fishing if resource status or market price are not good enough (adaptive management). However, in none of the fisheries, did users and government work together as equal partners, since authorities always have the final word on the decisions taken. Thus, we did not consider any of the barnacle fisheries to be cooperatively managed in sensu stricto, or in any other level above it (advisory, informative or self-governance, Table S4). Intermediate levels of co-management were found among the rest of the fisheries. In Morbihan, RNB and PNSACV intermediate levels between instructive and consultative were assigned, because even when under consultative co-managed, there are still instructional aspects in these fisheries, particularly in the way to achieve objectives.

#### Fisher’s participation

The participation of fishers ranged from passive (Portugal General) to interactive (Galicia) (Fig. 2), where users propose surveillance methods to control effort and actions for stock enhancement among others through management plans that are sent to fishing authorities for revision and eventual approval (Molares and Freire 2003). Also, the Galician authorities promote fishers’ participation providing funding support to *cofradías* to hire a fisheries biologist (a figure which is consistent with the Barefoot Ecologists concept (Prince 2003, 2010)), who gives management advice and facilitates communication between fishers and managers, researchers, surveillance officers and other stakeholders (Macho et al. 2013).

A functional participation was present in Asturias-West, Morbihan and RNB. In these fisheries, participation is mostly seen as a mean to achieve predetermine goals, and users are involved in surveillance activities and/or provide insights of resource status due to frequent research or regular meetings with the administration. In RNB, since 2018 there is a pilot program in place (CO-PESCA 2) to achieve a higher level of users’ participation in the fishery (see Table S11). Asturias-East, Orio and Bakio and PNSACV presented consultative participation, while in Portugal General and the Basque Country no exchange of information between users and the administration occurs. But as in the Basque Country harvesters have the option to meet managers upon request, so an intermediate level of participation between passive and consultative was assigned to the fishery.

#### Access structure

The access structure of the barnacle fisheries ranged from the least (open access in the Basque Country) to the most exclusive forms (TURFs in Galicia and Asturias-West) (Fig. 2).

In Galicia and Asturias-West, exclusive uses of fishing grounds are granted to fishers’ associations, who might restrict the quota, the number of harvesting days or close certain areas of their territory to reduce fishing effort. Fisheries of Morbihan, Asturias-East, Portugal General, RNB and PNSACV are subjected to a fixed number of licenses that endorse daily quotas (Table S15). However, we found a closer sense of ownership in RNB, where fishers, besides having daily quotas, are locally associated and have shown efforts towards the revalorization of the resource (participants focused on increasing the value of the product, which might indicate a reduction in the race-to-fish). The majority of fishers sell barnacles in batches depending on their size (Cruz et al. 2016), which might explain the higher revenues found in the fishery (Table 1) and there are current efforts towards the implementation of a certificate of origin for the fishery. Orio and Bakio were the only sites where an annual Total Allowable Catch (TAC) was found for the resource (Table S14), although it is not divided in quotas that guarantee a proportion of the catch. None of these plans set a maximum number of participants and the administration has the right to close the fishery once the TAC is reached. Hence, the race-to-fish typically seen under open access regimes is thought to occur.

### Sustainability assessment

**Fig. 3:**
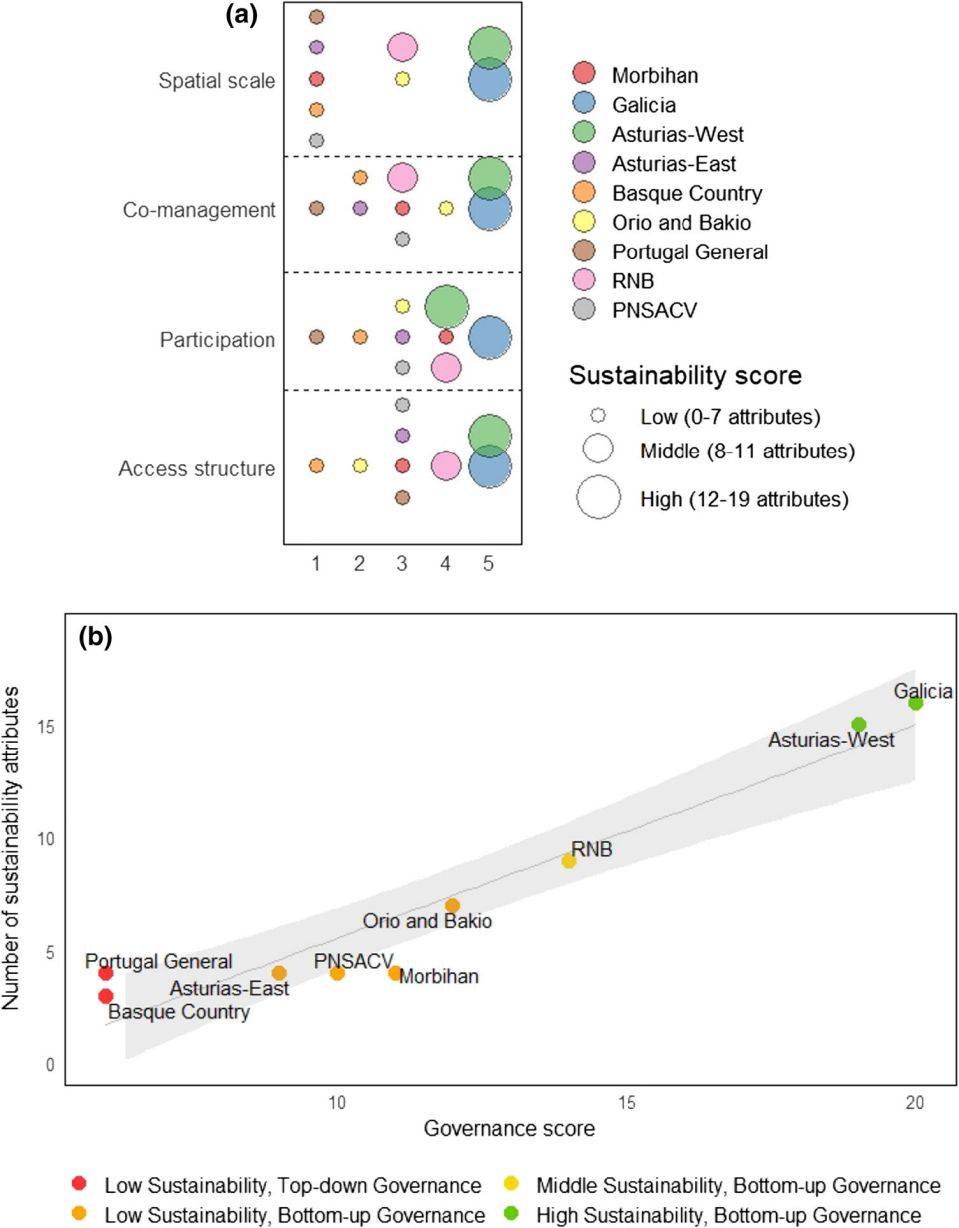
Figures show that governance score needs to surpass a certain threshold to accumulate many of the attributes that determine sustainability. **a** Spatial scale of management, co-management, fisher’s participation and access structure organized from lowest (1) to highest (5) levels (see corresponding levels in Fig. 2, note that for the spatial scale there are only 3 levels). Circle size indicates the number of sustainability attributes present per barnacle fishery. **b** Linear regression between the number of sustainability attributes and the governance score (*P-value* < 0.001***, Adjusted *R*^2^ = 0.899). Based on the sustainability and governance score four groups of fisheries are identified presented in different colors

The different sustainability attributes per stalked barnacle fishery were assessed (Table 2). Detailed explanations for each attribute are presented in the SSMM from Table S8 to S26. Barnacle fisheries ranged from displaying three (Basque Country General) to 16 (Galicia) attributes, with just two fisheries above 12 and thus, considered to have high sustainability scores (Galicia and Asturias-West). In the governance of these two fisheries the finest spatial detail in the management scale (patch) was found. Galicia and Asturias-West were also the only co-managed TURFs for barnacles in SW Europe, presenting higher levels of participation and exclusivity in the access structure (Fig. 3a).

**Table 2:** Sustainability attributes (Gutiérrez et al. 2011) present per stalked barnacle fishery in Southwest Europe. Attributes are grouped in the following categories: RS = resource system, RU = resource units, GS = governance system, U = users system

			Morbihan	Galicia	Asturias West	Asturias East	BCountry General	Orio and Bakio	Portugal General	RNB	PNASCV
Sustainability attributes	RS	Defined boundaries (S8)		√	√			√		√	
	RU	Sedentary/low mobility resource (S9)	√	√	√	√	√	√	√	√	√
	GS	Co-management in law (S10)		√	√						
	GS	Local authorities support (S11)	√	√	√			√		√	
	GS	Long-term management policy (S12)		√	√						
	GS	Scientific advice (S13)		√	√			√		√	
	GS	Monitoring control surveillance (S14)		√	√	√		√		√	
	GS	Global catch quotas (S15)						√			
	GS	Individual or community quotas (S16)	√	√	√	√			√	√	√
	GS	TURF (S17)		√	√						
	GS	Spatially explicit management (S18)		√	√						
	GS	Minimum size (S19)		√	√	√	√	√	√	√	√
	GS	Protected areas (S20)	√				√		√	√	√
	GS	Seeding or restocking (S21)									
	U	Social cohesion (S22)		√	√					√	
	U	Leadership (S23)		√	√						
	U	Self-enforcement (S24)		√	√						
	U	Tradition in self-organization (S25)		√							
	U	Influence in local market (S26)		√	√						

See Tables S8 to S26 in SSMM for a detailed explanation of each attribute

Among fisheries, a positive relationship between the number of sustainability attributes and governance score was found (*P-value* < 0.001***, Adjusted R^2^ = 0.899) (Fig. 3b), with the four governance elements showing a positive relationship with sustainability independently (Figure S2). Increases in the governance score not only led to overall higher sustainability levels, but to an even growth in the number of attributes of the governance and users’ systems (Figure S3). However, when the four governance elements were only present at middle or high levels, fisheries were considered moderately or highly sustainable (Fig. 3a). These findings suggest the need to surpass a certain threshold in the governance score to accumulate many of the attributes that determine sustainability.

We distinguish four different groups of fisheries based on their sustainability and governance scores (Fig. 3b). We found two bottom-up fisheries that presented high sustainability (Galicia and Asturias-West), and one bottom-up at an intermediate sustainability level (RNB). Among the six fisheries that displayed low sustainability, two of them were subjected to top-down governance (Basque Country and Portugal General), while the other four (Morbihan, Asturias-East, Orio and Bakio and PNSACV) had bottom-up settings.

## Discussion

Through the comparison of heterogeneous governance settings targeting the same resource (stalked barnacle), we found that nested spatial scales of management, co-management, active fisher’s participation and secure access rights promote social-ecological sustainability in small-scale fisheries (Fig. 3b). Although governance elements could in theory be independent from each other, our results show them to be mutually reinforcing and, in some instances, display synergistic effects (e.g., exclusive access rights allow for higher fisher’s participation). Furthermore, it is not the presence of the four governance elements that drives the sustainability of a fishery, but rather their level of implementation (Fig. 3a). Hence, efforts for effective governance should be placed in the accomplishment of a minimum combination of local scales of management, instructive-consultative co-management, functional participation and access rights through individual quotas (IQs). We suggest that surpassing that threshold across governance settings will start to adequately promote social, economic and ecological sustainability in small-scale fisheries.

Out of the nine stalked barnacle fisheries studied, only Galicia and Asturias-West (Spain) achieved high sustainability scores (Fig. 3b). These two fisheries are managed through TURFs where fishers actively participate in all aspects of the management and share responsibilities with the administration in the decision-making (Molares and Freire 2003; Rivera et al. 2014). Through strict access rights, fishers have the right to sanction and/or exclude access to users who do not comply with regulations; key in the success of other small-scale fisheries (particularly in sedentary or low mobility stocks) in Latin America (Orensanz and Seijo 2013; Defeo et al. 2016) and Japan (Yamamoto 1995). Facilitated by the sense of empowerment, Galicia and Asturias-West present a high involvement of fishers in data collection and assessment. In these fisheries, the creation of communities that act as mediating links between authorities and individuals, avoids the typical top-down role of governments and the consequent passive behaviour of fishers (Pomeroy et al. 2001; Jentoft 2005; Jentoft et al. 2010). Galicia and Asturias-West also present an adaptive spatial management with nested scales at regional, local and patch/rock level. This detailed spatial scale is only possible through the close collaboration between fishers and managers in these fisheries, exemplified in the classification of over 250 zones according to resource quality in Asturias-West in just 200 km of coast (Rivera et al. 2014).

The importance of the access structure, co-management and participation also explains the sustainability scores recorded in RNB (Portugal), although to a lower extent (Fig. 3b). The long-term licenses granted in this fishery (Jacinto et al. 2011) in combination with their particularly low accessibility (i.e., it is a group of islands), secures ownership rights to fishers and excludes others from access in a similar way that a TURF does. The higher extent of governance settings in RNB in comparison to the other Portuguese fisheries explains the more stable management (Cruz et al. 2015) and the higher proportion of barnacles with commercial value found there (Sousa et al. 2013). The rest of the fisheries scored low in sustainability, despite four of them (Orio and Bakio, Morbihan, Asturias-East, PNSACV) being subjected to bottom-up governance settings. The situation of unsustainable fisheries is particularly critical as the addition of new attributes will not promote their sustainability until at least eight of the attributes are reached (Gutiérrez et al. 2011), denoting the large amount of improvements needed to potentially reach it in the future.

Our results place the social dimension in the spotlight of small-scale governance, as the set of attributes related to the user system (i.e., leadership, social cohesion and self-enforcement among others) are key in distinguishing between sustainable and unsustainable fisheries (Table 2). This demonstrates that management should not only be focused on the technical rules per se, but more importantly, on the way these regulations are established. Most of the sustainability attributes from the user system need a complex set of conditions at individual/household, community and supra-community levels to occur (Pomeroy et al. 2001), making their implementation particularly complex. These attributes were only found in barnacle fisheries that scored high in governance (Galicia and Asturias-West), suggesting that when governance elements are implemented at low or middle extents, fisheries might need to undergo significant governance changes to achieve these attributes.

Stalked barnacles are managed based on administrative or political boundaries that do not account for the spatial structure of the resource. This mismatch between management and biology has often resulted in failure for other small-scale resources that are sedentary or with low mobility (Ouréns et al. 2015), as it divides local populations into territories subjected to different measures. Besides resource units, boundaries also define the fishing communities targeting them. In a group of fishers, ethnic and religious homogeneity, geographic proximity and linkages between “friends of friends” (i.e., triadic closure) promote community structure (Pomeroy et al. 2001; Alexander et al. 2018). That is the case of the Galician fishery, where fishers’ associations (*cofradías*) and management plans have been traditionally designed based on the social component to a greater degree than on the biological. However, when setting up small-scale fisheries boundaries, the extent to which social factors might compensate the inconsistency between management and biology remains unexplored.

Our work shows that unsustainability in small-scale fisheries is associated to inadequate governance structures, as previously pointed out in other studies (Hilborn et al. 2005; Orensanz et al. 2005; Prince 2010; Jentoft and Chuenpagdee 2015). The recent release of the Voluntary Guidelines for Securing Small-Scale Fisheries (known as SSF Guidelines) by FAO is a step toward overcoming the lack of social-ecological sustainability through adequate governability in the sector (FAO 2015). Nonetheless, putting the SSF Guidelines into action remains a challenge in numerous communities around the world (Singleton et al. 2017; Courtney et al. 2019). We believe that our study contributes to the correct implementation of several of the SSF Guidelines principles. Access structure through IQs and TURFs recognize and empower traditional forms of organization and support the collection of traditional knowledge, contributing to the implementation of the respect of cultures principle. Barnacle fisheries subjected to functional or interactive participation under consultative-cooperative co-management promote open data and well-defined policies in broadly understandable formats, contributing to the guidelines transparency. However, as the central focus of SSF Guidelines is in the context of food security and poverty alleviation in developing countries, other governance elements such as human rights and equity would need to be added to address all their principles and broaden the concept of sustainability beyond the present paper.

The work of by Gutiérrez et al. (2011) used to assess sustainability in our study has limitations. By focusing on the presence of a minimum number of attributes independently of their combination and context, this framework ignores characteristics known to alter fishery outcomes (Cinner and Huchery 2013). There are examples of fisheries where certain combinations of attributes and/or a particular context might have produced a desirable outcome, despite having a low number of sustainability attributes (Oliver et al. 2015). Although the limitations of Gutiérrez et al. (2011) should be acknowledged, a recent evaluation of 20 studies that have empirically applied the social-ecological system framework (Thiel et al. 2015) found that Gutiérrez et al. (2011) was the study where the most relevant variables were selected. Explicit definitions and reasons of their selection were provided (construct validity) at the same time that additional variables that are not part of the social-ecological framework were included (external validity). We believe these validity principles further supports our use of Gutiérrez et al. (2011) in our analysis.

Our results show that bottom-up governance promotes sustainability in small-scale fisheries, but that a broader consideration beyond the mere presence or absence of certain governance elements is crucial to effectively promote sustainability. Access structure through IQs and TURFs (level 4 and 5 in Fig. 3a) provide incentives for the involvement of fishers in management and decision-making. Functional and interactive participation (level 4 and 5 in Fig. 3a) and co-management between instructive and cooperative levels (level 3, 4 and 5 in Fig. 3a) support healthy stocks through the collection and use of fishers’ traditional knowledge (Prince 2003). This aspect is particularly important in data-poor fisheries like sedentary or low mobility stocks (Orensanz et al. 2005). In these situations, the use of fisher knowledge (usually broad qualitative information but spatially explicit) is particularly important to identify changes in catch trends (Johannes 1998; Prince 2010); especially in the context of threats like climate change. We consider the fisheries biologist present in most fishers’ associations in Galicia (a figure that is consistent with the concept of Barefoot Ecologists (Prince 2003, 2010)) to play a key role for the sustainability of the fishery. Facilitating the communication between fishers and managers, the fisheries biologist promotes the integration of traditional knowledge with general fisheries expertise (Macho et al. 2013), promoting the connection of the social and ecological dimensions of the fishery.

## Conclusion

The study of Gutiérrez et al. (2011) was fundamental in clarifying what is needed to achieve social, economic and ecological sustainability in small-scale and industrial fisheries, by highlighting the cumulative effect of 19 key attributes as the main drivers. The recent release of the Voluntary Guidelines for Securing Small-Scale Fisheries (SSF Guidelines) by FAO is a step forward to confront the lack of social-ecological sustainability through adequate governability in the sector. However, there is an important question to address this issue: what can be changed in the governance system to foster all those sustainability attributes? Our study provides insight by finding a direct relationship between the governance level and the sustainability attributes leading to fisheries success. Fisheries can upgrade on four key governance elements (the spatial scale of management, co-management, fisher’s participation and the access structure), since they need to surpass a certain threshold to accumulate many of the attributes that determine sustainability. This is not an easy task, but based on our case study, it is clear that fisheries agencies can start by first securing access rights in the long-term (by prioritizing the social dimension while promoting adaption to the local circumstances, especially in poverty alleviation and food security contexts, not examined here). This approach will give fishers an incentive to increase their participation in the monitoring and evaluation of the fishery, which can generate spatially explicit information to guide the decision-making process. Although other aspects highlighted in the SSF Guidelines that were not taken into account in this work and that are particularly important in developing countries (e.g., human rights and dignity, equity and equality, etc.) should be carefully considered, the governance elements addressed in this study (i.e., the access structure, nested spatial scales of management, fisher’s participation and co-management) are still very relevant to fisheries sustainability in both developing and developed countries.

## Supplementary Information

Below is the link to the electronic supplementary material.

Supplementary material 1 (PDF 1386 kb)
