# Supplementary material for: Establishing a governance threshold in small-scale fisheries to achieve sustainability
Source: Ambio. 2021 Aug 17;51(3):652–65. doi: 10.1007/s13280-021-01606-x (PMC8800988; doi:10.1007/s13280-021-01606-x)
Supplement: Supplementary file 1 — Supplementary material 1 (PDF 1386 kb) [file 13280_2021_1606_MOESM1_ESM.pdf]

Ambio

Electronic Supplementary Material
This supplementary material has not been peer reviewed.

Title: Establishing a governance threshold in small-scale fisheries to achieve sustainability

Alba Aguión, Elena Ojea, Lucía García-Flórez, Teresa Cruz, Joxe Mikel Garmendia, Dominique Davoult, Henrique Queiroga, Antonella Rivera, José Luis Acuña-Fernández, Gonzalo Macho

Figure S1. Relationship between the governance score and the levels of the four governance elements analyzed per stalked barnacle fishery in Southwest Europe. Governance elements include the spatial scale of management (see levels in Table S3), co-management (Table S4), fisher's participation (Table S5) and the access structure (Table S6).

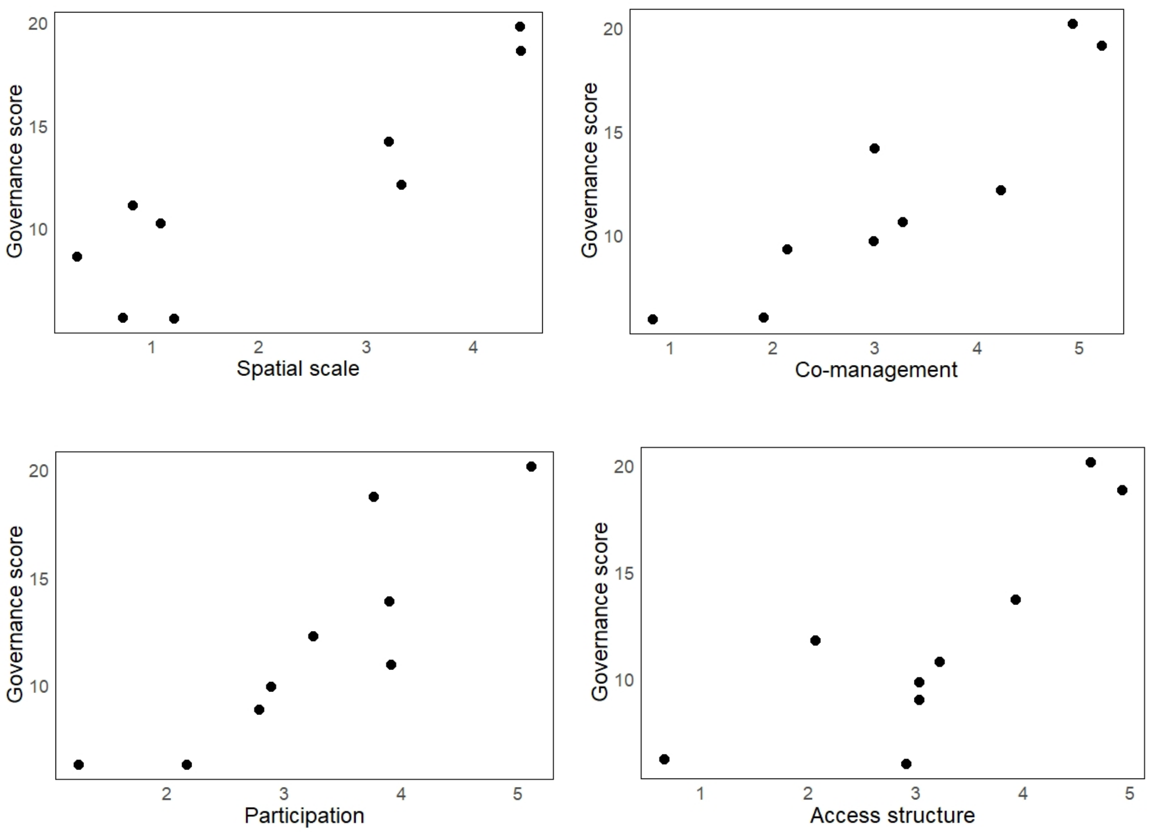

Figure S2. Relationship between the number of sustainability attributes and the levels of the four governance elements analyzed per stalked barnacle fishery in Southwest Europe. Governance elements include the spatial scale of management (see levels in Table S3), co-management (Table S4), fisher's participation (Table S5) and the access structure (Table S6).

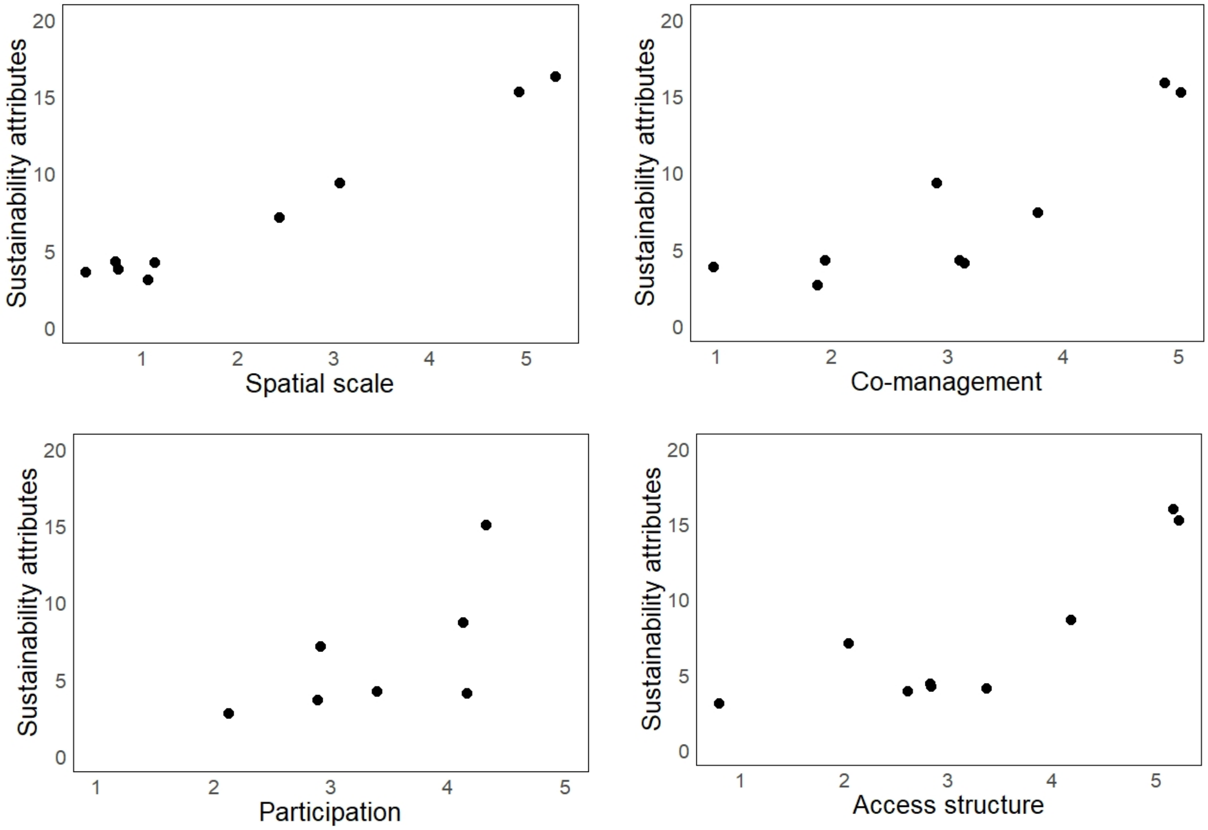

Figure S3. Presence of sustainability attributes per group (GS= governance system, U= users system) per stalked barnacle fishery in Southwest Europe.

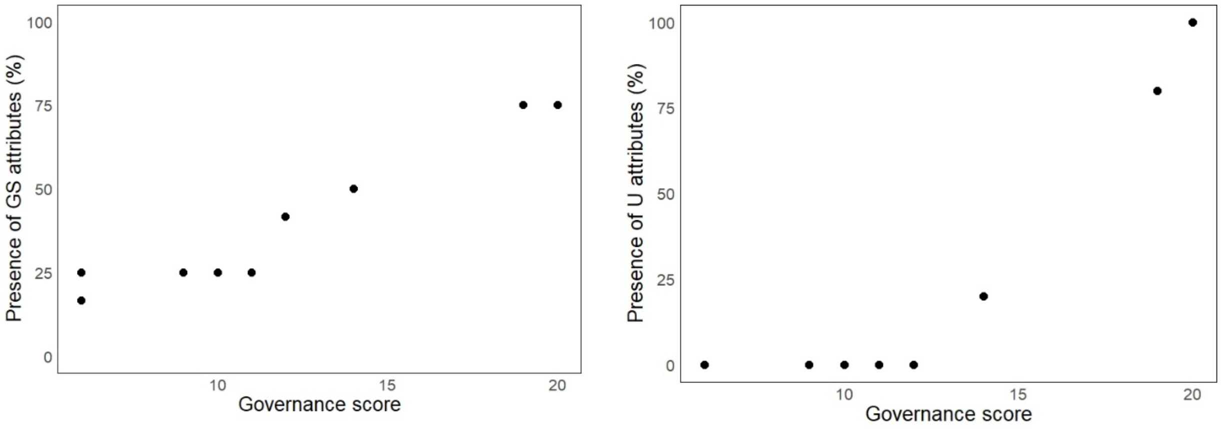

Table S1. Responsible bodies of the nine stalked barnacle fisheries analyzed in the study. Responsible bodies are the ultimate institutions on charge of the political, economic and administrative authority of the resource.

Fishery	*Responsible body*	*Level*
Morbihan	Comité National des Pêches Maritimes et des Élevages Marins (CNPMEM)	National
Galicia	Dirección Xeral de Pesca, Acuicultura e Innovación Tecnolóxica da Xunta de Galicia	Regional
Asturias-West	Dirección General de Pesca Marítima del Principado de Asturias	Regional
Asturias-East	Dirección General de Pesca Marítima del Principado de Asturias	Regional
Basque Country General	Dirección de Pesca y Acuicultura del Gobierno Vasco	Regional
Orio and Bakio	Dirección de Pesca y Acuicultura del Gobierno Vasco	Regional
Portugal General	Direção-Geral de Recursos Naturais, Segurança e Serviços Marítimos (DGRM)	National
Reserva Natural das Berlengas (RNB)	Direção-Geral de Recursos Naturais, Segurança e Serviços Marítimos (DGRM), Instituto da Conservação da Natureza e das Florestas (ICNF)	National
Parque Natural do Sudoeste Alentejano e Costa Vicentina (PNSACV)	Direção-Geral de Recursos Naturais, Segurança e Serviços Marítimos (DGRM), Instituto da Conservação da Natureza e das Florestas (ICNF)	National

Table S2. Data used for the socioeconomic characterization of the stalked barnacle fisheries in terms of number of fishers, landings (volume and value) and ex-vessel price (Table 1).

Fishery	*Fishers*	*Landings volume*	*Landings value and price*
Morbihan	Active<br>2013-2016<br>CNPMEM	Reported landings<br>2013-2016<br>CNPMEM	No data<br>CNPMEM and fishers estimate
Galicia	Active. On boat fishers estimated assuming 2 fishers per active boat<br>2013-2016<br>Xunta de Galicia	Reported landings<br>2013-2016<br>Xunta de Galicia	Reported value<br>2013-2016<br>Xunta de Galicia
Asturias-West	Active<br>2013-2016<br>Centro Experimentación Pesquera (CEP)	Reported landings<br>2013-2016<br>Centro Experimentación Pesquera (CEP)	Market data 2013-2016<br>Centro Experimentación Pesquera (CEP)
Asturias-East	Active<br>2013-2016<br>Centro Experimentación Pesquera (CEP)	Reported landings<br>2013-2016<br>Centro Experimentación Pesquera (CEP)	Market data 2013-2016<br>Centro Experimentación Pesquera (CEP)
Basque Country General	-	-	-
Orio and Bakio	Active<br>2013-2016<br>AZTI	Reported landings<br>2013-2016<br>AZTI	-
Portugal General and the combination of Portugal General, RNB and PNSACV	Active<br>2008-2014<br>DGRM<br>(seen in Carvalho et al.2017)	Reported landings<br>2008-2014<br>DOCAPESCA, 2016<br>(seen in Carvalho et al. 2017)	Reported value<br>2008-2014<br>DOCAPESCA, 2016<br>(seen in Carvalho et al. 2017)
Reserva Natural das Berlengas (RNB)	Number of licenses<br>2012 (Sousa et al. 2013)	Reported landings<br>2008-2012<br>(Cruz et al. 2015)	Estimate by interviews to professional fishers 2013<br>(Cruz et al. 2016)
Parque Natural do Sudoeste Alentejano e Costa Vicentina (PNSACV)	Number of licenses<br>2012 (Sousa et al. 2013)	-	Estimate by interviews to professional fishers 2013<br>(Cruz et al. 2016)

Table S3. Broad spatial scales recognized for the biology and management of small-scale benthic resources (Orensanz et al. 2016). Scales are ordered from a higher to a lower degree of spatial detail.

Spatial scale	*Description*
**Microscale (Patch)**	Neighborhoods within local populations, usually corresponding to small portions of the fishing bed. Defined in the order of 1 kilometer
**Mesoscale (Local)**	At the level of local populations, usually corresponding to the scale of fishing beds. Defined in the order of 10s kilometers, but below the mean larval dispersal distance
**Macroscale (Regional)**	Aggregate of local populations, defined in the order of 10s-100s kilometers. Average larval dispersal of stalked barnacles has been estimated in 100 km using an Individual-Based Model (maximum pelagic larval duration was set in 60 days and several larval behavior scenarios were used) coupled to a hydrodynamic model (Nolasco, Dubert and Queiroga, unpublished data). Both models have been successfully tested before in other species in the same area (Domingues et al. 2012; Gomes et al. 2016)

Table S4. Self-governance, co-management levels (Sen and Nielsen 1996) and centralized management fisheries description. Regimes are ordered from a higher responsibility level of fishers in the decision-making to a gradually decreasing one.

Fisheries regime		*Description*
**Self-governance***		Government has done a total power devolution to the local users (e.g., traditional marine tenure systems)
**Co-management**	**Informative**	Government has delegated authority to user groups, who are responsible for informing on the decisions taken
	**Advisory**	Users advice government on the decisions to be taken and government endorses them
	**Cooperative**	Government and users cooperate together as equal partners in decision-making
	**Consultative**	Mechanisms exists for governments to consult with users but all decisions are taken by government
	**Instructive**	Minimal exchange of information between government and users. Government informs the users on the decisions to be taken
**Centralized management**		Absence of mechanisms to dialogue with users

* Some authors include self-governance or community-based management as a type of co-management

Table S5. Description of participatory levels ordered from a higher participation of users to a gradually decreasing one. Fisher's participation is focused on the involvement of users in the monitoring, control and surveillance. Classification was originally designed for agriculture (modified from Pretty (1995).

Level of participation	*Description*
**Self-mobilization**	Users participate by taking initiatives independently of external institutions to change systems. Users develop contacts with external institutions for resources and technical advice they need, but retain control over how resources are used
**Interactive participation**	Industry driven partnership. Participation is a right, not just a just a mean to achieve goals. Often, industry produces independent management advice and voluntarily participates in monitoring design, data collection and analysis
**Functional participation**	Government driven partnership. Participation seen as a mean to achieve predetermine goals. Users might participate by forming groups to meet predetermined objectives related to the project. Such involvement tends to arise only after major decisions have already been made by external agents
**Participation for material incentives**	People participate by contributing resources, for example, labour, in return for food, cash or other material incentives. People have no stake in prolonging practices when incentives end
**Participation by consultation**	Users participate by being consulted or by answering questions. External agents define problems and information gathering processes, and so control analysis
**Passive participation**	People participate by being told what has been decided or has already happened. Unilateral announcements by administration

Table S6. The access structure ordered from the most to the least exclusive forms (Hilborn et al. 2005).

Level of access	*Description*
**TURF**	Long-term ownership rights over an area and its resources to particular users group excluding others from access. Rights that in some cases may come close to ownership
**IQs (Individual Quotas)**	The licenses granted do not only allow access but contain a proportion of the total catch or effort. IQs might be transferable or not. The most significant element is that by specifying what proportion of the resource each participant may catch, the race-to-fish is eliminated. The participants can then concentrate on reducing the costs of fishing and increasing the value of the product, both of which lead to increases in profitability
**Limited entry**	There is usually a fixed number of licenses issued and to participate in the fishery one must either purchase a license from an existing participant or receive one through for example, a merits ranking. In limited entry fisheries there is no guarantee of catch, licenses simply permit the right to participate. Licenses might include a maximum catch per fisher to reduce harvest
**Open access**	Any individual wishing to go fishing can do so, often with the purchase of a license for a nominal fee. Regulations of a gear and season are often introduced to reduce the level of harvest. Most commercial fisheries go through this period at their initial stages

Table S7. Attributes known to promote long-term sustainability in co-managed fisheries (Gutiérrez et al. 2011). Definition, group (RS= resource system, RU= resource units, GS= governance system, U= users system) and the potential direct/indirect effects of each attribute are provided. For each attribute a reference to the table that justifies its presence or absence across the stalked barnacle fisheries in SW Europe is included.

Long-term sustainability attributes				
Group	***Name***	***Definition*** *Gutiérrez et al. 2011*	***Potential direct/indirect effects*** *Gutiérrez et al. 2011*	***Table***
RS	**Defined boundaries**	Clearly defined geographic boundaries (e.g., lakes, coastal lagoons, fjords)	Facilitates protection against outsiders, restricts fishermen dynamics, improves users communication, decreases monitoring effort and costs, increases ecological knowledge. Well-defined boundaries favor the implementation of self-policing strategies and a voluntary cooperative action to avoid infringement of rules	S8
RU	**Sedentary or low mobility resource**	Comprises sessile, sedentary and reduced mobility adult stages species with limited behavioral responses to stimuli	Facilitates targeting rights and responsibilities and local and spatially-explicit management, easier access in welldefined areas and easier monitoring and enforcement	S9
GS	**Co-management in law**	Co-management is supported by laws and decrees in the National Constitution	Gives users and their institutions the legal right to participate in the co-management process through management plans, enforcement of rules, etc.	S10
GS	**Local authorities support**	Local government encourages, supports, and participates in the comanagement process	Facilitates the process of implementation of comanagement at the local level	S11
GS	**Long-term management policy**	Refers to sustainability in time and stability of management plans and/or management institutions	Implementation of a long-term policy in a comanagement context generates a great incentive to fishers to adhere to and get involved with enforcing regulations, thus reducing the probability of occurrence of free-riders, illegal fishing, and short-term, profit maximizing behaviors	S12
GS	**Scientific advice**	Implies scientific advice and participation of Universities, NGOs or governmental institutions in the implementation of the co-management system	Scientific knowledge and advice on the ecology and resilience of targeted stocks play important roles in guiding co-management policies and governance development processes. Quality and quantity of information is improved through cooperation and information flow	S13
GS	**Monitoring, control and surveillance**	Fishery control, monitoring and surveillance by co-management authorities/institutions	Favors reliable information flow from fishers to policy makers, lowering monitoring, enforcement and transaction costs, and providing continuous fine-grained signals about resource status (adaptive co-management)	S14
GS	**Global catch quotas**	Resources are managed through assignment of global catch quotas (e.g. TACs)	Reinforces co-management if allocated together with other management tools in a context of management redundancy. Requires legislation and enforcement of legal frameworks, and cooperation of fisher-communities, which need to be adapted to countries and idiosyncrasies	S15

GS	**Individual or community quotas**	Resources are managed through individual, transferable or not, or community fishing quotas designed and implemented within the co-management regime	Creates incentives to self-management, self-enforcement and community empowerment	S16
GS	**TURF**	Formal Territorial Users Rights of Fishing	Generates a sense of exclusive use and ownership among fishers, who perceive they are receiving the equivalent of a “land grant” which has the form of a highly productive aquatic area	S17
GS	**Spatially explicit management**	Separate areas of management and/or spatially-explicit tools (e.g., rotational harvest strategies)	Enhances the probability of co-management success, particularly in spatially-structured stocks with low mobility, where the spatial distribution patterns of abundance are heterogeneous, and the spatial dynamics of the fishing process follows closely spatial variations in abundance at the scale of small sub-areas	S18
GS	**Minimum size**	Minimum size regulations, through mesh sizes, traps, hooks, etc	Reduces fishing mortality of undesired individual sizes and increases survival of spawning stocks. Particularly useful under co-management regimes when implemented with the active participation of fishers, promoting compliance with regulations	S19
GS	**Protected areas**	Formal no-take areas, marine reserves and/or protected areas with a considerable degree of fishermen/communities involvement (community-based reserves)	Enhances fisheries management and conservation of biodiversity, particularly in multi-species or on sedentary stocks, or for which broader ecological impacts of fishing are an issue. Successful use of protected areas in a comanagement context required in this study a case-by-case understanding of the spatial structure of impacted fisheries, ecosystems and human communities.	S20
GS	**Seeding or restocking**	Includes low-cost stock enhancement activities such as extensive culture, natural restocking or transplanting	Enhances stock productivity and population replenishment	S21
U	**Social cohesion**	Social cohesion including unity, trust, harmony, communication and cooperation given by common interests among users (e.g., effective participation of most community members in meetings). Generally related with community homogeneity	Enhances user’s cooperation, conflict resolution, collaboration with external partners, ability to exclude outsiders, and willingness to report rules breaking. Increases awareness and promotes co-management sustainability	S22
U	**Leadership**	Key influential users with entrepreneurial skills, highly motivated, respected as local leaders, and directly involved in management decisions	Promotes local self-organization, influences enforcement and rules compliance, alleviates attitudes towards destructive practices and helps conflict resolution. Improves communication, teamwork and systems thinking skills	S23
U	**Self-enforcement**	User’s ability and effectiveness in enforcing management regulations (e.g., clear and effective system of penalties imposed by strong operational rules specified, enforced and controlled by local fishers)	Encourages compliance on regulations resulting from management measures imposed in each co-managed site by the communities themselves, in agreement with the fishery management authorities, in order to sustain catch levels over	S24

			time	
U	**Tradition in self-organization**	History and tradition in self-organization and self-governance. Traditional social hierarchies and local institutions (e.g. native, religious, etc.)	May facilitate implementation of co-management when fisher communities have taken the responsibility for managing resources, often building upon old or traditional roots that include strong community rules	S25
U	**Influence in local market**	Users have influence in fish trading, rules and price control mechanisms	Co-management alters the power relations of different players, promoting shorter marketing chains and mitigating deleterious middlemen effects on economic returns perceived by fishers	S26

Table S8. Defined boundaries.

Fishery	*Presence/Absence*	*Description*	*Source*
Morbihan	Absence	-	
Galicia	Presence	Restrictive boundaries contained in 37 management plans assigned to 33 fishers' associations or *cofradías* (several *cofradías* have more than one management plan for the resource)	Pesca de Galicia 2019
Asturias-West	Presence	Restrictive boundaries contained in 8 management plans assigned to 12 fishers' associations or *cofradías* (some *cofradías* share management plans)	BOPA No.185 25-IX-2019
Asturias-East	Absence	-	
Orio and Bakio	Presence	Restrictive boundaries contained in 2 management plans recently approved (after an experimental phase since 2012)	BOPV No.96 23-V-2019<br><br>BOPV No.127 30-VI-2020
Basque General Regulation	Absence	-	
Portugal General	Absence	-	
Reserva Natural das Berlengas (RNB)	Presence	Restrictive boundaries contained in 1 management plan assigned to one fishers' association	Own expertise
Parque Natural do Sudoeste Alentejano e Costa Vicentina (PNSACV)	Absence	-	

Table S9. Sedentary resource.

Fishery	*Presence/Absence*	*Description*
Morbihan	Presence	Target stock (stalked barnacle) is a sessile intertidal crustacean
Galicia	Presence	Target stock (stalked barnacle) is a sessile intertidal crustacean
Asturias-West	Presence	Target stock (stalked barnacle) is a sessile intertidal crustacean
Asturias-East	Presence	Target stock (stalked barnacle) is a sessile intertidal crustacean
Orio and Bakio	Presence	Target stock (stalked barnacle) is a sessile intertidal crustacean
Basque General Regulation	Presence	Target stock (stalked barnacle) is a sessile intertidal crustacean
Portugal General	Presence	Target stock (stalked barnacle) is a sessile intertidal crustacean
Reserva Natural das Berlengas (RNB)	Presence	Target stock (stalked barnacle) is a sessile intertidal crustacean
Parque Natural do Sudoeste Alentejano e Costa Vicentina (PNSACV)	Presence	Target stock (stalked barnacle) is a sessile intertidal crustacean

Table S10. Co-management in law.

Fishery	***Presence/Absence***	***Description***	***Source***
Morbihan	Absence	-	
Galicia	Presence	Law states that harvesting can only be conducted under the umbrella of a management plan developed by a fishers' association or *cofradía* for its assigned territory. When the content of the plan is not adjusted to shellfish regulations, entails a resource subexploitation or does not align the biological criteria of the administration, managers have the right to modify the plan to achieve a more efficient resource use	DOGA No.13 20-I-1994<br><br>DOGA No.56 21-III-2000
Asturias-West	Presence	Law states that management plans are elaborated by the administration in collaboration with the fishers' associations or *cofradías* with the aim to preserve the resource and improve their commercialization. Plans are defined as agreements made between the administration and *cofradías* after subsequent modifications	BOPA No.185 25-IX-2019
Asturias-East	Absence	-	
Orio and Bakio	Absence	-	
Basque General Regulation	Absence	-	
Portugal General	Absence	-	
Reserva Natural das Berlengas (RNB)	Absence	-	
Parque Natural do Sudoeste Alentejano e Costa Vicentina (PNSACV)	Absence	-	

Table S11. Local authorities support.

Fishery	*Presence/Absence*	*Description*	*Source*
Morbihan	Presence	The National Committee responsible of the fishery (CNPMEM) has delegated authority to the Regional Committee (CRPMEM du Morbihan), from where arrangements such as the harvesting calendar are annually subjected to vote among fishers	JORF No.0157 10-VII-2018<br><br>Own expertise
Galicia	Presence	Management plans are actively developed by the fishers' associations or *cofradías* with the advice of a technical assistant (i.e., biologist), who is hired by the *cofradía* with the financial support of the local government	Macho et al. 2013
Asturias-West	Presence	Management plans are designed by the local government in close collaboration with the fishers' associations or *cofradías*	BOPA No.185 25-IX-2019
Asturias-East	Absence	-	
Orio and Bakio	Presence	Implementation of 2 management plans in an attempt to mimic the close collaboration between the administration and fishers occurring in Galicia and Asturias-West. Project "Proyecto piloto para el estudio de la viabilidad en la aplicación de planes de explotación del percebe en la costa del País Vasco" coordinated by AZTI-Tecnalia with local authorities support	Own expertise
Basque General Regulation	Absence	-	
Portugal General	Absence	-	
Reserva Natural das Berlengas (RNB)	Presence	Implementation of the pilot project CO-PESCA 2 (2018-2020) with the objective to operationalize a co-management committee and provide it with the tools to allow their continuity once the project finishes. Project CO-PESCA 2 coordinated by MARE-Instituto Politécnico de Leiria in collaboration with WWF (through ANP in Portugal), MARE-University of Évora and Instituto da Conservação da Natureza e das Florestas (ICNF), and including local authorities	Own expertise
Parque Natural do Sudoeste Alentejano e Costa Vicentina (PNSACV)	Absence	-	

Table S12. Long-term management policy.

Fishery	*Presence/Absence*	*Description*	*Source*
Morbihan	Absence	-	
Galicia	Presence	Licenses granted are annually suggested by the fishers' associations or *cofradías*, although it cannot exceed a number above from which harvesters do not obtain the minimum wage law. Harvesters annually renew their permits as long as they follow the requirements specified on them (if any); although by law, on foot harvesters have to work at least 70% of the harvesting days to be qualified for a renewal	DOGA No.56 21-III-2000
Asturias-West	Presence	Licenses granted are annually suggested by the fishers' associations or *cofradías*. Harvesters annually renew their permits as long as they follow the requirements specified on them (if any); although by law, harvesters have to assist at least from 10 or 20 (depending on the case) harvesting days to be qualified for a renewal	Rivera et al. 2014<br><br>BOPA No.185 25-IX-2019
Asturias-East	Absence	-	
Orio and Bakio	Absence	-	
Basque General Regulation	Absence	-	
Portugal General	Absence	-	
Reserva Natural das Berlengas (RNB)	Absence	-	
Parque Natural do Sudoeste Alentejano e Costa Vicentina (PNSACV)	Absence	-	

Table S13. Scientific advice.

Fishery	***Presence/Absence***	***Description***	***Source***
Morbihan	Absence	-	
Galicia	Presence	Most fishers' associations or *cofradías* count with scientific advice on a daily basis due to the presence of a technical assistant (i.e., biologist) that provides daily management advice to harvesters and has a key role in the design of the management plans	Macho et al. 2013
Asturias-West	Presence	Management arrangements, such as the establishment of temporal closures, are specifically proposed to each fishers' association or *cofradía* by authorities based on annual monitoring data (and approved with the agreement of *cofradías*)	Rivera et al. 2014
Asturias-East	Absence	-	
Orio and Bakio	Presence	At least one annual meeting between harvesters, managers and scientists is held to decide on fishery arrangements based on monitoring data	Own expertise
Basque General Regulation	Absence	-	
Portugal General	Absence	-	
Reserva Natural das Berlengas (RNB)	Presence	Scientific advice has been occasionally involved in decision-making. Examples include in 2011 the cease of the rotational schemes and the reduction of the resource size limit asked by the harvesters with support from the scientists. Participation of institutions that provide scientific advice in a project that promotes the implementation of co-management (see Table S10)	Own expertise
Parque Natural do Sudoeste Alentejano e Costa Vicentina (PNSACV)	Absence	-	

Table S14. Monitoring, control and surveillance.

Fishery	***Presence/Absence***	***Description***	***Source***
Morbihan	Absence	-	
Galicia	Presence	Annual monitoring of catches, effort, CPUE and sales per fishers' association or *cofradía*. In some *cofradía*s monitoring of percentage coverage, density estimates and/or mean landing size. Insights of resource status thanks to the daily exchange of information between fishers and the technical assistant (i.e. biologist) of the *cofradía*. Control and surveillance done at a regional (Galicia) and local (*cofradía*) level	Molares and Freire 2003<br><br>Macho et al. 2013
Asturias-West	Presence	Annual monitoring of catches, effort, CPUE and sales in several areas per fishers' association. Summer monitoring of percentage coverage and stock biomass for a different fishers' association each year. Government hires one surveillance officer per management plan that is on charge of controlling harvesters' catch everyday that the activity occurs	Rivera et al. 2014<br><br>Own expertise
Asturias-East	Presence	Annual monitoring of catches, effort, CPUE and sales. Harvesters are provided a logbook to register catches. Summer monitoring of percentage coverage and stock biomass, although usually not annually conducted	Own expertise
Orio and Bakio	Presence	Annual monitoring of catches. Harvesters are provided a logbook to register catches. Annual monitoring to estimate mean density, weight and size of the resource over a dozen of stations at each management plan	Own expertise
Basque General Regulation	Absence	-	
Portugal General	Absence	-	
Reserva Natural das Berlengas (RNB)	Presence	Annual monitoring of catches and insights of resource status due to frequent research involving interviews with fishers and technical assessments. Monitoring and surveillance is done by institutions that promote the implementation of co-management	Own expertise
Parque Natural do Sudoeste Alentejano e Costa Vicentina (PNSACV)	Absence	-	

Table S15. Global catch quotas.

Fishery	*Presence/Absence*	*Description*	*Source*
Morbihan	Absence	-	
Galicia	Absence	-	
Asturias-West	Absence	-	
Asturias-East	Absence	-	
Orio and Bakio	Presence	Annual global quota that changes annually/biannually based on monitoring data<br><br>Global catch quotas of 1,985 kg in Orio and 2,000 kg in Bakio	BOPV No.96 23-V-2019<br><br>BOPV No.127 30-VI-2020
Basque General Regulation	Absence	-	
Portugal General	Absence	-	
Reserva Natural das Berlengas (RNB)	Absence	-	
Parque Natural do Sudoeste Alentejano e Costa Vicentina (PNSACV)	Absence	-	

Table S16. Individual or community quotas.

Fishery	*Presence/Absence*	*Description*	*Source*
Morbihan	Presence	120 kg per fisher per day	CRPMEM Bretagne Decision No.191-2017
Galicia	Presence	3 to 10 kg per fisher per day depending on the management plan, harvesting modality (on foot vs. on boat), and time of the year	Pesca de Galicia 2019
Asturias-West	Presence	5 to 8 kg per fisher per day depending on the management plan and time of the year	BOPA No.185 25-IX-2019
Asturias-East	Presence	5 to 8 kg per fisher per day depending on the time of the year	BOPA No.185 25-IX-2019
Orio and Bakio	Absence	-	
Basque General Regulation	Absence	-	
Portugal General	Presence	20 kg per fisher per day	Sousa et al. 2013
Reserva Natural das Berlengas (RNB)	Presence	20 kg per fisher per day	Sousa et al. 2013
Parque Natural do Sudoeste Alentejano e Costa Vicentina (PNSACV)	Presence	10 to 15 kg per fisher per day depending on the time of the year	Sousa et al. 2013

Table S17. TURF.

Fishery	*Presence/Absence*	*Description*	*Source*
Morbihan	Absence	-	
Galicia	Presence	System where management plans are spatially allocated to fishers' guilds or *cofradías*	Molares and Freire 2003
Asturias-West	Presence	System where management plans are spatially allocated to fishers' guilds or *cofradías*	Rivera et al. 2014
Asturias-East	Absence	-	
Orio and Bakio	Absence	-	
Basque General Regulation	Absence	-	
Portugal General	Absence	-	
Reserva Natural das Berlengas (RNB)	Absence	-	
Parque Natural do Sudoeste Alentejano e Costa Vicentina (PNSACV)	Absence	-	

Table S18. Spatially explicit management.

Fishery	*Presence/Absence*	*Description*	*Source*
Morbihan	Absence	-	
Galicia	Presence	Over half of the fishers' associations or *cofradías* subdivide their areas in usually between 3 and 5 zones. Total or partial closures are established within each zone during the year to promote stock recovery and maximize economic revenues (best quality areas are opened in the high season)	Own expertise
Asturias-West	Presence	Subdivision of the area of the fishers' associations or *cofradías* in 267 zones. Total or partial closures are established within each zone during the year to promote stock recovery and maximize economic revenues (best quality areas are opened in the high season)	Rivera et al. 2014
Asturias-East	Absence	-	
Orio and Bakio	Absence	-	
Basque General Regulation	Absence	-	
Portugal General	Absence	-	
Reserva Natural das Berlengas (RNB)	Absence	-	
Parque Natural do Sudoeste Alentejano e Costa Vicentina (PNSACV)	Absence	-	

Table S19. Minimum size. Owing to the particular morphology of stalked barnacles there are different ways to measure the individuals, usually using the calcareous plates that conform its triangular carapace or capitulum. The different morphological variables found in the European fisheries are RC (rostro carina length), DBC (diameter of the base of the capitulum) and TL (total length). DBC and TL have been converted into RC respectively (Parada et al. 2013; Parada et al. 2012) .

Fishery	***Presence/Absence***	***Description***	***Source***
Morbihan	Absence	-	
Galicia	Presence	18.3 mm (RC) on 60% of the catch<br>Original biometric: 15 mm (DBC)	DOGA No.226<br>27-XI-2012
Asturias-West	Presence	18 mm (RC) on 60% of the catch	BOPA No.185<br>25-IX-2019
Asturias-East	Presence	18 mm (RC) on 60% of the catch	BOPA No.185<br>25-IX-2019
Orio and Bakio	Presence	13.4 mm (RC) on 60% of the catch<br>Original biometric: 40 mm (TL)<br>without stretching	BOPV No.96<br>23-V-2019<br><br>BOPV No.127<br>30-VI-2020
Basque General Regulation	Presence	13.4 mm (RC) on 60% of the catch<br>Original biometric: 40 mm (TL)<br>without stretching	BOPV No.94<br>20-V-1997
Portugal General	Presence	20 mm (RC) on 75% of the catch	Sousa et al. 2013
Reserva Natural das Berlengas (RNB)	Presence	23 mm (RC) on 50% of the catch	Sousa et al. 2013
Parque Natural do Sudoeste Alentejano e Costa Vicentina (PNSACV)	Presence	20 mm (RC) on 75% of the catch	Sousa et al. 2013

Table S20. Protected areas.

Fishery	*Presence/Absence*	*Description*	*Source*
Morbihan	Presence	2 no-take areas in Groix Island	CRPMEM Bretagne Carte 17
Galicia	Absence	-	
Asturias-West	Absence	-	
Asturias-East	Absence	-	
Orio and Bakio	Absence	-	
Basque General Regulation	Presence	Gaztelugatxe Marine Reserve (no-take area)	Borja et al. 2006
Portugal General	Presence	Luis Saldanha Marine Park (no-take)	Sousa et al. 2013
Reserva Natural das Berlengas (RNB)	Presence	Permanent no-take areas	Sousa et al. 2013
Parque Natural do Sudoeste Alentejano e Costa Vicentina (PNSACV)	Presence	Permanent no-take areas	Sousa et al. 2013

Table S21. Seeding or restocking.

Fishery	*Presence/Absence*	*Description*
Morbihan	Absence	There is no seeding or restocking practice for stalked barnacles
Galicia	Absence	There is no seeding or restocking practice for stalked barnacles
Asturias-West	Absence	There is no seeding or restocking practice for stalked barnacles
Asturias-East	Absence	There is no seeding or restocking practice for stalked barnacles
Orio and Bakio	Absence	There is no seeding or restocking practice for stalked barnacles
Basque General Regulation	Absence	There is no seeding or restocking practice for stalked barnacles
Portugal General	Absence	There is no seeding or restocking practice for stalked barnacles
Reserva Natural das Berlengas (RNB)	Absence	There is no seeding or restocking practice for stalked barnacles
Parque Natural do Sudoeste Alentejano e Costa Vicentina (PNSACV)	Absence	There is no seeding or restocking practice for stalked barnacles

Table S22. Social cohesion.

Fishery	***Presence/Absence***	***Description***	***Source***
Morbihan	Absence	-	
Galicia	Presence	Fishers are organized in 33 fishers' associations or *cofradías* with an assigned territory. From *cofradías* fishers usually participate and speak in a united voice, sharing relatively homogeneous interests in the resource	Molares and Freire 2003
Asturias-West	Presence	Fishers are organized in 12 fishers' associations or *cofradías* with an assigned territory. *Cofradías* conduct regular meetings to determine the status of the management plan and zones. Fishers share relatively homogeneous interests in the resource and propose in a united voice changes in the measures when needed	Rivera et al. 2014
Asturias-East	Absence	-	
Orio and Bakio	Absence	-	
Basque General Regulation	Absence	-	
Portugal General	Absence	-	
Reserva Natural das Berlengas (RNB)	Presence	Fishers belong to one fishers' association allocated to the territory of RNB and conduct regular meetings to discuss about the management plan or other fishery issues	Cruz et al. 2015<br><br>Own expertise
Parque Natural do Sudoeste Alentejano e Costa Vicentina (PNSACV)	Absence	-	

Table S23. Leadership.

Fishery	***Presence/Absence***	***Description***	***Source***
Morbihan	Absence	-	
Galicia	Presence	Fishers' associations or *cofradías* count with a *junta general* (i.e., general shareholders meetings), responsible of the decision-making. In addition, fishers usually aggregate in *agrupaciones sectoriales* (i.e., sectoral working groups) to promote an efficient extraction and commercialization of the resource. Fishers leaders are in charge every year of the design and daily implementation (in collaboration with technical assistants of the *cofradías*) of a very adaptive management plan	Own expertise
Asturias-West	Presence	Fishers' associations or *cofradías* count with a *junta directiva* (i.e., general shareholders meetings), responsible of the decision-making. Fishers that are highly motivated usually collaborate in decision-making process with the *junta directiva*	Rivera et al. 2019<br><br>Own expertise
Asturias-East	Absence	-	
Orio and Bakio	Absence	-	
Basque General Regulation	Absence	-	
Portugal General	Absence	-	
Reserva Natural das Berlengas (RNB)	Absence	-	
Parque Natural do Sudoeste Alentejano e Costa Vicentina (PNSACV)	Absence	-	

Table S24. Self-enforcement.

Fishery	*Presence/Absence*	*Description*	*Source*
Morbihan	Absence	-	
Galicia	Presence	Fishers invest their own resources in the enforcement of the resource by setting a local surveillance services (marine guards directly hired by the *cofradías* but co-paid between fishers/*cofradía* and fisheries administration), collaborating with governmental officers to prevent poaching. Fishers also participate in the enforcement activities personally. However, users often declare that many cases go unpunished or with small fines	Molares and Freire 2003<br><br>Macho et al. 2013<br><br>Own expertise
Asturias-West	Presence	Fishers occasionally carry out surveillance in special interest areas. In 3 of the TURFs, all users have agreed on personally carrying out surveillance. However, according to users many cases go unpunished or with small fines	Rivera et al. 2014
Asturias-East	Absence	-	
Orio and Bakio	Absence	-	
Basque General Regulation	Absence	-	
Portugal General	Absence	-	
Reserva Natural das Berlengas (RNB)	Absence	-	
Parque Natural do Sudoeste Alentejano e Costa Vicentina (PNSACV)	Absence	-	

Table S25. Tradition in self-organization.

Fishery	*Presence/Absence*	*Description*	*Source*
Morbihan	Absence	-	
Galicia	Presence	Harvesters have been organized in local fishers' associations or *cofradías* since Middle Ages, which had religious bases. Traditional exploitation of the resource in the region	Taboada 2007
Asturias-West	Absence	-	
Asturias-East	Absence	-	
Orio and Bakio	Absence	-	
Basque General Regulation	Absence	-	
Portugal General	Absence	-	
Reserva Natural das Berlengas (RNB)	Absence	-	
Parque Natural do Sudoeste Alentejano e Costa Vicentina (PNSACV)	Absence	-	

Table S26. Influence in the local market.

Fishery	*Presence/Absence*	*Description*	*Source*
Morbihan	Absence	-	
Galicia	Presence	Most fishers' associations or *cofradías* have the capacity to commercialize the catch, managing the first sale markets. Fishers' guilds that conduct spatially explicit management open the best quality areas during the high season (i.e., summer and Christmas) ensuring that good quality barnacles are sold at its highest price. In order to increase the price, fishers regularly decide not to go fishing, or not to sell, when prices are considered too low	Molares and Freire 2003<br><br>Own expertise
Asturias-West	Presence	Most fishers' associations or *cofradías* have the capacity to commercialize the catch, managing the first sale markets. Through the establishment of spatially explicit management the best quality areas are open during the high season (i.e., Christmas) ensuring that good quality barnacles are sold at its highest price	Rivera et al. 2014
Asturias-East	Absence	-	
Orio and Bakio	Absence	-	
Basque General Regulation	Absence	-	
Portugal General	Absence	-	
Reserva Natural das Berlengas (RNB)	Absence	-	
Parque Natural do Sudoeste Alentejano e Costa Vicentina (PNSACV)	Absence	-	

References

BOPA No. 185 25-IX-2019, Resolución 18 Septiembre 2019. Consejería de Desarrollo Rural, Agroganadería y Pesca de Asturias. https://sede.asturias.es/bopa/disposiciones/repositorio/LEGISLACION43/66/3/001U0063CQ0001.pdf.

BOPV No. 94 20-V-1997, Decreto 102/1997 6 Mayo 1997. Consejera de Desarrollo Económico e Infraestructuras del País Vasco. https://www.euskadi.eus/y22-bopv/es/bopv2/datos/1997/05/9702594a.pdf.

BOPV No. 96 23-V-2019, Orden 7 Mayo 2019. Consejera de Desarrollo Económico e Infraestructuras del País Vasco. https://opendata.euskadi.eus/catalogo/contenidos/orden/bopv201902453/es_def/index.shtml.

BOPV No. 127 30-VI-2020, Orden 10 Junio 2020. Consejera de Desarrollo Económico e Infraestructuras del País Vasco. https://www.euskadi.eus/gobierno-vasco/-/eli/es-pv/o/2020/06/10/(7)/dof/spa/html/.

Borja, A., I. Muxika, and J. Bald. 2006. Protection of the goose barnacle *Pollicipes pollicipes* Gmelin, 1790 population: the Gaztelugatxe Marine Reserve (Basque Country, northern Spain). *Scientia Marina* 70(2): 235-242. https://doi.org/10.3989/scimar.2006.70n2235.

Carvalho, A.N., P. Vasconcelos, D. Piló, F. Pereira, and M.B. Gaspar. 2017. Socio-economic, operational and technical characterization of gooseneck barnacle (*Pollicipes pollicipes*) in SW Portugal: Insights towards fishery co-management. *Marine Policy* 78: 34-44. https://doi.org/10.1016/j.marpol.2017.01.008.

CRPMEM Bretagne Decision No. 191-2017 Du 28 Decembre 2017. Comité regional des pêches maritimes et des élevages marins de Bretagne. http://www.cdpmem56.fr/wp-content/uploads/2018/01/191-2017-pouces-pieds-56-2018.pdf.

CRPMEM Bretagne, Carte 17 Peche des Pocues-Pieds. Comité regional des pêches maritimes et des élevages marins de Bretagne. https://doc.terramaris.fr/CRPMEM/Cartes/CARTE_017_poucepied_2013.pdf.

Cruz, T., D. Jacinto, A. Sousa, N. Penteado, D. Pereira, J.N. Fernandes, T. Silva, and Castro, J.J. 2015. The state of the fishery, conservation and management of the stalked barnacle *Pollicipes pollicipes* in Portugal. *Marine Environmental Research* 112(B): 73-80. .

Cruz, T., D. Jacinto, A. Sousa, N. Penteado, D. Pereira, J.N. Fernandes, T. Silva, and Castro, J.J. 2016. PERCEBES - Gestão, Ecologia e Conservação do Percebe em Portugal. Relatório final do projeto piloto 31-03-05-FEP-11. Universidade de Évora, Laboratório de Ciências do Mar e MARE - Centro de Ciências do Mar e do Ambiente.

DOGA No. 13 20-I-1994, Decreto 423/1993 17 Diciembre 1994. Consellería de Pesca, Marisquero y Acuicultura, Xunta de Galicia. https://www.xunta.gal/diario-oficial-galicia/mostrarContenido.do?lang=es&paginaCompleta=false&rutaRelativa=true&idEstado=5&ruta=/1994/19940120/SeccionesE9A_es.html.

DOGA No. 56 21-III-2000, Orden 6 Marzo 2000. Consellería de Pesca, Marisquero y Acuicultura, Xunta de Galicia. https://www.xunta.gal/dog/Publicados/2000/20000321/Anuncio6E1A_es.html.

DOGA No. 226 27-XI-2012, Orden 27 Julio 2012. Consellería de Pesca, Marisquero y Acuicultura, Xunta de Galicia. https://www.xunta.gal/dog/Publicados/2012/20121127/AnuncioG0165-191112-0006_es.pdf.

Domingues, C. P., R. Nolasco, J. Dubert, and H.Queiroga. 2012. Model-Derived Dispersal Pathways from Multiple Source Populations Explain Variability of Invertebrate Larval Supply. *PLoS ONE* 7: e35794. https://doi.org/10.1371/journal.pone.0035794.

Gomes, I., L.G. Peteiro, R. Albuquerque, R. Nolasco, J. Dubert, S.E. Swearer, and H. Queiroga. 2016. Wandering mussels: using natural tags to identify connectivity patterns among Marine Protected Areas. *Marine Ecology Progress Series* 552:159-176. https://doi.org/10.3354/meps11753.

Gutiérrez, N.L., R. Hilborn, and O. Defeo. 2011. Leadership, social capital and incentives promote successful fisheries. *Nature* 470: 386-389. https://doi.org/10.1038/nature09689.

Hilborn, R., J.M. Orensanz, and A.M. Parma. 2005. Institutions, incentives and the future of fisheries. *Philosophical Transactions of The Royal Society B Biological Sciences* 360(1453): 47-57. https://doi.org/10.1098/rstb.2004.1569.

JORF No. 0157 10-VII-2018, Délibération B42/2018. Comité national des pêches maritimes et des élevages marins de la République française. .

Macho, G., I. Naya, J. Freire, S. Villasante, and J. Molares. 2013. The Key Role of the *Barefoot Fisheries Advisors* in the Co-managed TURF System of Galicia (NW Spain). *AMBIO A Journal of the Human Environment* 42(8): 1057-1069. https://doi.org/10.1007/s13280-013-0460-0.

Molares, J., and J. Freire. 2003. Development and perspectives for community-based management of the gooseneck barnacle (*Pollicipes pollicipes*) fisheries in Galicia (NW Spain). *Fisheries Research 65(1-3*): 485-492. http://doi.org/10.1016/j.fishres.2003.09.034.

Orensanz, J.M., A.M. Parma, and S.J. Smith. 2016. Dynamics, Assessment, and Management of Exploited Natural Scallop Populations. In *Scallops: Biology, Ecology, Aquaculture, and Fisheries,* ed. S.E. Shumway, and G.J. Peterson, 611-695. Oxford: Elsevier Science.

Pesca de Galicia. 2019. Data from “Plans de xestión para recursos específicos 2019”. Consellería do Mar, Xunta de Galicia. www.pescadegalicia.gal.

Parada, J.M., E. Iglesias, R. Outeiral, and J. Molares. 2013. Diameter of the base of the capitulum as a biometric variable of the goose barnacle *Pollicipes pollicipes* (Gmelin, 1789) (Cirripedia, Lepadomorpha). *Crustaceana* 86: 1527-1538. https://doi.org/10.1163/15685403-00003251.

Parada, J.M., R. Outeiral, E. Iglesias, and J. Molares. 2012. Assessment of goose barnacle (*Pollicipes pollicipes* Gmelin, 1789) stocks in management plans: design of a sampling program based on the harvesters’ experience. *ICES Journal of Marine Science* 69(10): 1840-1849. https://doi.org/10.1093/icesjms/fss157.

Pretty, J.N. 1995. Participatory Learning For Sustainable Agriculture. *World Development* 23(8): 1247-1263. https://doi.org/10.1016/0305-750X(95)00046-F.

Rivera, A., S. Gelcich, L. García-Florez, and J.L. Acuña. 2019. Social attributes can driver or deter the sustainability of bottom-up management systems. *Science of the Total Environment* 690: 760-767. https://doi.org/10.1016/j.scitotenv.2019.06.323.

Rivera, A., S. Gelcich, L. García-Flórez, J.L. Alcázar, and J.L. Acuña. 2014. Co-management in Europe: Insights from the gooseneck barnacle fishery in Asturias, Spain. *Marine Policy* 50(A): 300-308. https://doi.org/10.1016/j.marpol.2014.07.011.

Taboada, M.S. 2017. Las prácticas contables de las cofradías de pescadores Gallegas. La coerción como vehículo de institucionalización cultural normativa. *Revista Galega de Economía* 16.

Sen, S., and J.R. Nielsen. 1996. Fisheries co-management: a comparative analysis. *Marine Policy* 20(5): 405-418. https://doi.org/10.1016/0308-597X(96)00028-0.

Sousa, A., D. Jacinto, N. Penteado, P. Martins, J. Fernandes, T. Silva, J.J. Castro, and T. Cruz. 2013. Patterns of distribution and abundance of the stalked barnacle (*Pollicipes pollicipes*) in the central and southwest coast of continental Portugal. *Journal of Sea Research* 83: 187-194. https://doi.org/10.1016/j.seares.2013.04.005.
